# Biochemical Characterizations of Human TMPK Mutations Identified in Patients with Severe Microcephaly: Single Amino Acid Substitutions Impair Dimerization and Abolish Their Catalytic Activity

**DOI:** 10.1021/acsomega.1c05288

**Published:** 2021-12-06

**Authors:** Junmei Hu Frisk, Jo M. Vanoevelen, Jörgen Bierau, Gunnar Pejler, Staffan Eriksson, Liya Wang

**Affiliations:** † Department of Anatomy, Physiology and Biochemistry, Swedish University of Agricultural Sciences, Uppsala SE-750 07, Sweden; ‡ Department of Clinical Genetics, Maastricht University Medical Centre+ and GROW School for Oncology and Developmental Biology, Maastricht 6202 AZ, The Netherlands; § Department of Medical Biochemistry and Microbiology, Uppsala University, Uppsala SE-750 07, Sweden

## Abstract

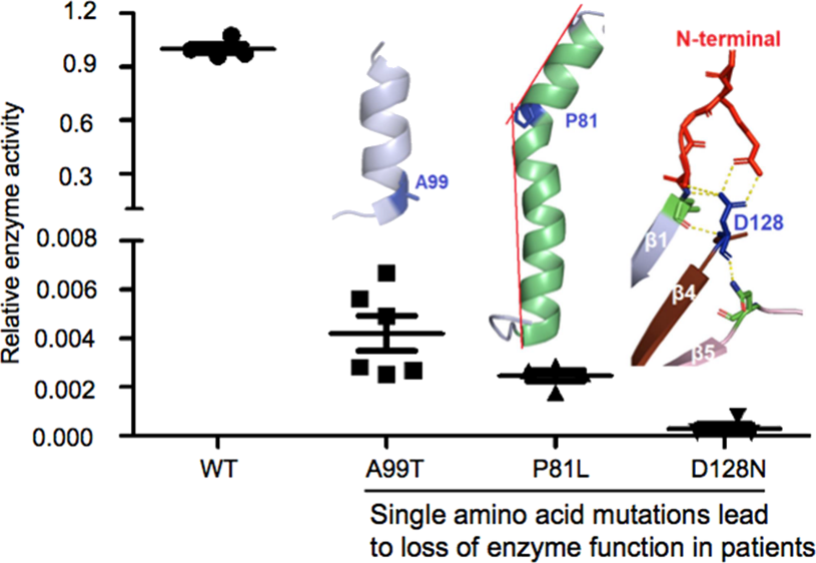

Deoxythymidylate kinase (TMPK) is a key enzyme in the synthesis of deoxythymidine triphosphate (dTTP). Four TMPK variants (P81L, A99T, D128N, and a frameshift) have been identified in human patients who suffered from severe neurodegenerative diseases. However, the impact of these mutations on TMPK function has not been clarified. Here we show that in fibroblasts derived from a patient, the P81L and D128N mutations led to a complete loss of TMPK activity in mitochondria and extremely low and unstable TMPK activity in cytosol. Despite the lack of TMPK activity, the patient-derived fibroblasts apparently grew normal. To investigate the impact of the mutations on the enzyme function, the mutant TMPKs were expressed, purified, and characterized. The wild-type TMPK mainly exists as a dimer with high substrate binding affinity, that is, low *K*_M_ value and high catalytic efficiency, that is, *k*_cat_/*K*_M_. In contrast, all mutants were present as monomers with dramatically reduced substrate binding affinity and catalytic efficiencies. Based on the human TMPK structure, none of the mutated amino acids interacted directly with the substrates. By structural analysis, we could explain why the respective amino acid substitutions could drastically alter the enzyme structure and catalytic function. In conclusion, TMPK mutations identified in patients represent loss of function mutations but surprisingly the proliferation rate of the patient-derived fibroblasts was normal, suggesting the existence of an alternative and hitherto unknown compensatory TMPK-like enzyme for dTTP synthesis. Further studies of the TMPK enzymes will help to elucidate the role of TMPK in neuropathology.

## Introduction

Deoxynucleotide triphosphates (dNTPs) are essential building blocks for DNA synthesis. The synthesis of deoxythymidine triphosphate (dTTP) is accomplished by the de novo and salvage pathways. In the salvage pathway, thymidine kinase 1 (TK1, in cytosol) and thymidine kinase 2 (TK2, in mitochondria) phosphorylate thymidine (dT) to thymidine monophosphate (dTMP). In the de novo pathway, thymidylate synthase converts deoxyuridine monophosphate to dTMP in the presence of tetrahydrofolate. dTMP is then further phosphorylated to deoxythymidine diphosphate (dTDP) by deoxythymidylate kinase (TMPK) (EC 2.7.4.9). The final phosphorylation step from dTDP to dTTP is catalyzed by nonspecific nucleoside diphosphate kinases. Thus, TMPK is the bottleneck of dTTP synthesis since it is essential for both the de novo and salvage pathways of dTTP synthesis.^1^

In humans, *DTYMK* encodes TMPK and recently four *DTYMK* variants have been identified in human patients who suffered from severe congenital neurodegenerative diseases. In one study, compound heterozygous mutations (P81L and D128N) were found in one patient and a homozygous mutation (P81L) in another patient. Both patients suffered from severe neurodevelopment disorders with a vanishing brain syndrome and died at 18 and 32 months of age, respectively.^2^ In another study, two siblings with compound heterozygous mutations (34 bp deletion causing frameshift and a missense A99T mutation) were reported, and the patients also suffered from neurodevelopmental disorders, severe microcephaly, hypotonia, and severe intellectual disability. Still, they were alive at 2 respective 7 years of age at the time of study. The authors suggested that the frameshift and A99T mutations may lead to loss of TMPK function and mitochondrial DNA depletion, albeit no experimental evidence was presented.^3^

To help to understand the role of TMPK in neurodegenerative disorders, we have here investigated the impact of the P81L and D128N mutations on the proliferation, dTTP synthesis capacity, and TMPK activity in patient-derived fibroblasts. We also expressed and characterized all three missense TMPK mutants identified in human patients in order to clarify the effects of these point mutations at the structural and functional levels. Finally, TMPK structure analysis helped to explain why these amino acid substitutions result in drastically reduced substrate binding affinity and catalytic activity and impaired dimerization.

## Results

### P81L and D128N Compound Heterozygous Mutations Do Not Impair the Proliferation of the Fibroblasts Derived from a Patient

**Figure 1:**
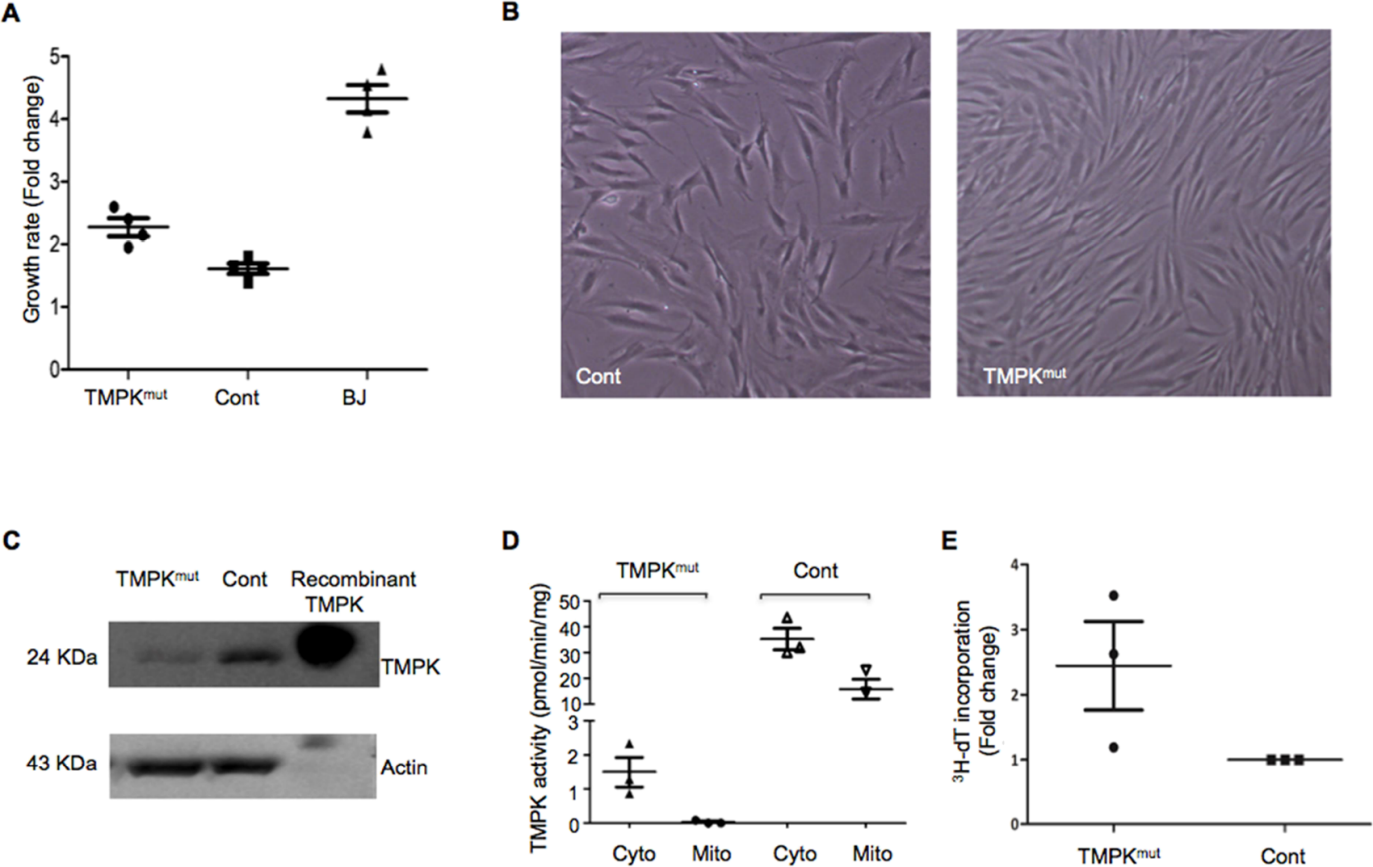
Characterization of TMPK^mut^ fibroblasts. (A) Growth rate of TMPK^mut^, Cont, and immortal fibroblasts (BJ). The same number of cells were seeded and incubated for 3 days and then the cells were trypsinized and counted. The results represent fold changes in cell numbers during the 3-day incubation period; (B) comparison of the morphology of TMPK^mut^ and Cont cells. Approximately 1.5 million cells were seeded simultaneously; TMPK^mut^ reached 90% confluency faster than Cont. The TMPK^mut^ cells exhibited an elongated shaped compared with Cont. (C) Western blot analysis of the TMPK expression. Extracts from TMPK^mut^ and Cont cells were used; equal amounts of protein were loaded. Beta-actin was used a loading control; (D) cytosolic and mitochondrial TMPK activities; and (E) incorporation of ^3^H-dT into DNA in TMPK^mut^ fibroblasts. DNA was extracted from TMPK^mut^ and control (Cont) fibroblasts after 10 h of incubation with ^3^H-dT and the radioactivity was counted.

Considering the key role of TMPK in dTTP synthesis, we first asked whether the growth rate of primary fibroblasts derived from the patient might be impaired. Patient-derived fibroblasts (TMPK^mut^), control fibroblasts (Cont), and a fibroblast cell line (BJ) were cultured under the same conditions and the number of cells was quantified. In the course of 1 month, the growth rate of TMPK^mut^ fibroblasts was clearly higher than that of Cont but lower than that of the BJ (Figure 1A). TMPK^mut^ cells attached to the culture flask displayed elongated shapes 6 h after seeding and reached 90% confluence faster than the Cont cells (Figure 1B). The proliferation capability of TMPK^mut^ diminished gradually after 2 months and stopped after 3 months, while the proliferation of Cont stopped in less than 2 months. Taken together, these data indicate that the mutations in the *DTYMK* gene do not impair the proliferation of the patient-derived fibroblasts.

### TMPK Protein and Activity Levels and Subcellular Localization Are Affected in TMPK^mut^ Fibroblasts

The expression of the TMPK protein in cell lysates from both TMPK^mut^ and Cont was analyzed by Western blot using a human TMPK-specific antibody. As shown in Figure 1C, the level of TMPK protein in TMPK^mut^ fibroblasts was lower than that in the control cells. The TMPK activity in cytosolic and mitochondrial preparations was also measured using ^3^H-dTMP as the substrate. In TMPK^mut^ cells, no TMPK activity could be detected in the mitochondria and low TMPK activity was detected in the cytosol, while in control cells, TMPK activity was detected both in the mitochondria and cytosol (Figure 1D). We also measured the thymidine kinase 1 (TK1) activity, a marker for cell proliferation, and found that TK1 activity in TMPK^mut^ fibroblasts was significantly higher than that in control cells (Figure S1), which was in-line with the higher proliferation rate observed for the TMPK^mut^ cells.

### ^3^H-dT Uptake and Metabolism in TMPK^mut^ and Control Cells

The extremely low TMPK activity and fast growth of the TMPK^mut^ cells promoted us to investigate the dTTP synthesis using ^3^H-dT (tritium-labeled thymidine). Stepwise phosphorylation of ^3^H-dT into ^3^H-dTTP and incorporation of ^3^H-dT into DNA can be used as a measure for TMPK activity since the salvage pathway also requires TMPK for dTTP synthesis. As shown in Table 1, after 10 h of incubation with ^3^H-dT in the culture medium, dTMP (68%) accounted for the highest percentage of ^3^H-labeled nucleotides among the soluble nucleotides extracted from control cells. In TMPK^mut^ cells, dTDP and dTTP (52%) were the most dominant nucleotides, indicating that the TMPK^mut^ cells have higher capacity to convert dTMP to dTDP. Furthermore, the percentage of ^3^H-dT in control cells was also higher than that in TMPK^mut^ cells, and this could be explained by the higher TK1 activity in the TMPK^mut^ cells (Figure S1). In the culture media, after removal of the cells, >82% of the radioactivity remains as ^3^H-dT for both types of cells. We could also detect labeled dTMP, dTDP, and dTTP in the media, which may be secreted by the cells or released from dead cells. In agreement with the intracellular ^3^H-dT metabolism, in DNA extracted from TMPK^mut^ cells, the extent of radiolabeling was also higher than that of controls (Figure 1E). These results suggested that there is an alternative and previously unknown TMPK-like enzyme in TMPK^mut^ cells for dTTP synthesis and its high capability to synthesize dTTP may explain the observed higher growth rate.

**Table 1:** Distribution of ^3^H-dT Nucleotides in Soluble Nucleotide Extracts and Media^a^

	extracts		media	
	Cont (%)	TMPK^mut^ (%)	Cont (%)	TMPK^mut^ (%)
dT	6	3	82	83
dTMP	68	39	6	5
dTDP + dTTP	24	52	4	5

a Data are shown as the percentage of total radioactivity recovered in extracts or media.

### Factors Affecting TMPK Activity in TMPK^mut^ Cell Extracts

**Figure 2:**
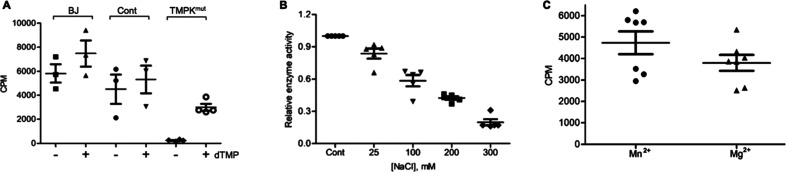
Factors affecting TMPK activity. (A) Effect of dTMP in the lysis buffer. Freshly harvested cells (0.2 million of each cell type) were homogenized in buffer ± 0.1 μM dTMP, and then equal amounts of protein were used for TMPK activity measurements (shown as CPM). (B) Effect of NaCl on the TMPK activity from TMPK^mut^ cells; TMPK activity at different NaCl concentrations relative to controls without NaCl (as 1.0). (C) Effect of Mn^2+^ and Mg^2+^. Extracts containing equal amounts of proteins were used in the TMPK activity measurements in the presence of 4 mM Mg^2+^ or Mn^2+^ ions. The results are shown as CPM.

The higher capacity of the TMPK^mut^ cells to synthesize dTTP raised the question as to whether there is a second TMPK-like enzyme that compensates for the lack of TMPK activity. Earlier studies have shown that TMPK is unstable in cell lysates and dTMP was needed to stabilize the TMPK activity.^4,5^ Therefore, a pairwise comparison of the effect of dTMP on TMPK activity in cell lysates was conducted. As shown in Figure 2A, addition of dTMP to cell lysates resulted in higher TMPK activity in TMPK^mut^ cells. In contrast, the TMPK activity was relatively stable regardless of the presence of dTMP in control and BJ cell extracts (Figure 2A). The TMPK activity from TMPK^mut^ cells was also sensitive to the presence of salts such as NaCl in the lysis buffer. As shown in Figure 2B, there was a concentration-dependent reduction of TMPK activity in TMPK^mut^ cell lysates in the presence of NaCl. Since divalent metal ions are important cofactors for TMPK, we assayed TMPK^mut^ cell extracts in the presence of different divalent metal ions such as Mg^2+^, Mn^2+^, Co^2+^, Zn^2+^, and Ca^2+^ and found that only Mg^2+^ and Mn^2+^ could serve as cofactors for the enzyme. The highest TMPK activity in TMPK^mut^ cells was detected in the presence of Mn^2+^ (Figure 2C), while in control cells, the TMPK activity and recombinant human TMPK activity were higher in the presence of Mg^2+^ (data not shown), as was reported earlier.^5^

### Molecular Characterization of the Wild-Type (WT) and Mutant TMPKs

**Figure 3:**
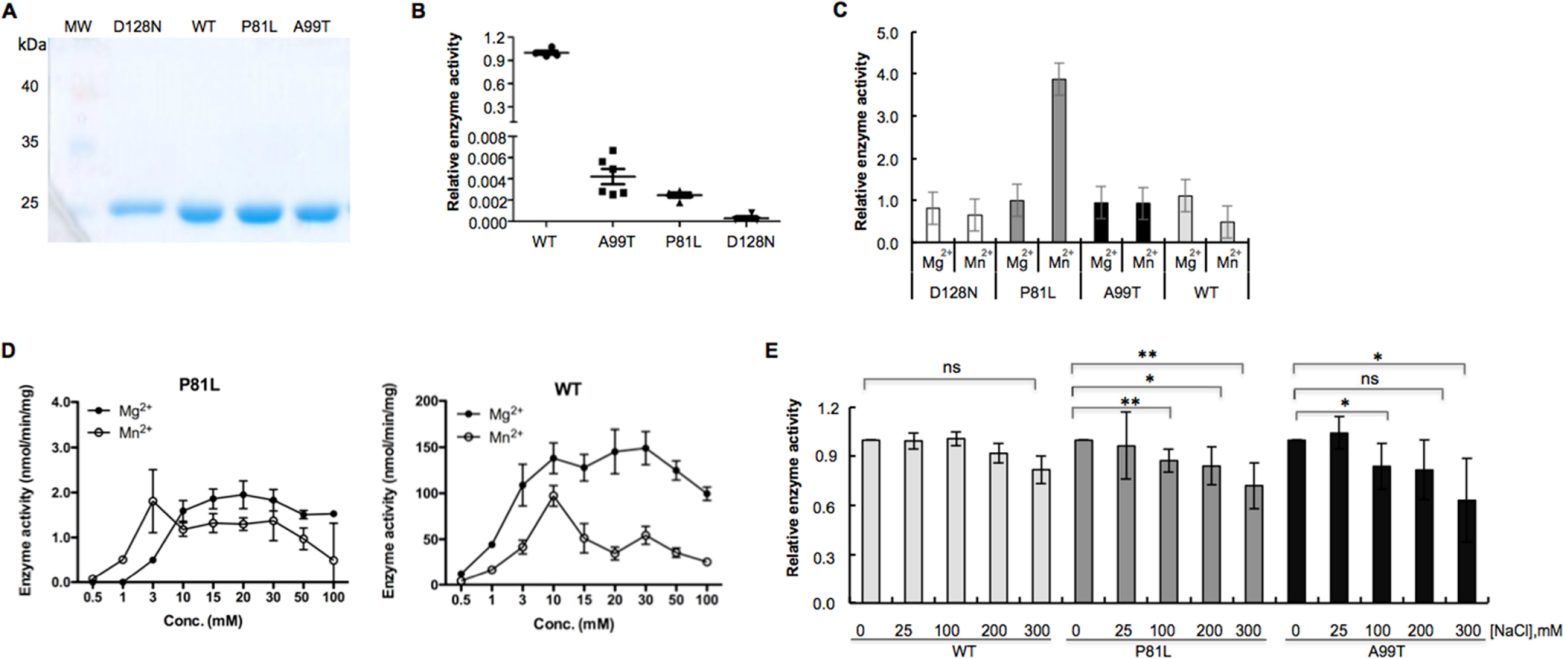
Expression and characterization of mutant TMPKs. (A) SDS-PAGE analysis of purified WT, P81L, A99T, and D128N mutant TMPK; (B) specific activity of mutant TMPK compared with that of the WT. Relative activity is shown (WT as 1.0); (C) effects of Mg^2+^ and Mn^2+^ on the TMPK activity. For each enzyme, the activity was normalized to that with Mg^2+^ (as 1.0); (D) titration of Mn^2+^ and Mg^2+^ with P81L mutant TMPK. ATP and dTMP concentrations were kept constant, 1 mM and 2 μM, respectively; and (E) effect of NaCl on WT and mutant TMPKs.

To study the impact of the respective mutations on TMPK function, all three TMPK mutants and WT TMPK were expressed in *Escherichia coli* (*E. coli*), and the recombinant enzymes were affinity-purified to >95% purity as judged by sodium dodecyl sulfate–polyacrylamide gel electrophoresis (SDS-PAGE) analysis (Figure 3A). We then compared the mutant and WT enzymes and found that the specific activity was substantially reduced in all three mutants ranging from 0.08 to 0.4% of that of the WT enzyme (Figure 3B).

### Steady-State Kinetic Analysis

To provide further insight into the effects of the respective TMPK mutations on enzyme catalysis, a steady-state kinetic analysis was conducted. The WT enzyme showed high binding affinity for both dTMP and adenosine triphosphate (ATP) with *K*_M_ values of 1.75 and 1.11 μM, respectively. In contrast, the A99T and P81L mutant enzymes had remarkably low binding affinity for both substrates, with *K*_M_ values for dTMP 14- and 66-fold higher, respectively, than those of the WT enzyme. Moreover, the *K*_M_ value for ATP was ∼40-fold higher than that of the WT enzyme. Both the A99T and P81L mutants had also significantly lower catalytic efficiency (*k*_cat_/*K*_M_) compared with that of WT TMPK (Table 2).

**Table 2:** Kinetic Parameters of Mutants and WT TMPK^a^

	dTMP			ATP		
	*K*_M_ (μM)	*k*_cat_ (s^–1^)	*k*_cat_/*K*_M_ (M^–1^ s^–1^)	*K*_M_ (μM)	*k*_cat_ (s^–1^)	*k*_cat_/*K*_M_ (M^–1^ s^–1^)
WT	1.75 ± 0.88	3.24 ± 0.23	1.85 × 10^6^	1.11 ± 0.15	2.78 ± 0.03	2.51 × 10^6^
A99T	24.6 ± 5.4	6.92 ± 0.42	0.28 × 10^6^	41.3 ± 4.03	4.82 ± 0.07	0.11 × 10^6^
P81L	115.9 ± 31.2	17.2 ± 2.13	0.14 × 10^6^	43.1 ± 3.67	5.39 ± 0.07	0.12 × 10^6^

a Activity determination was performed at 21 °C using a coupled spectrophotometric assay. Each measurement was repeated five times, and the data are given as mean ± SD. The data were fitted to the Michaelis–Menten equation using GraphPad Prism.

The D128N mutant TMPK had too low activity to allow a reliable kinetic analysis. As shown in Figure S2, the D128N mutant activity, at variable dTMP and ATP concentrations, did not follow typical enzyme kinetics, and therefore, no *K*_M_ or *k*_cat_ was calculated from these data.

### Effects of Mg^2+^, Mn^2+^, and Salt

Since the TMPK activity detected in the TMPK^mut^ cells was sensitive to both divalent metal ions and salt, we investigated the effects of these on the recombinant mutant enzymes. Of the three mutants, only the P81L mutant responded positively to the switch from Mg^2+^ to Mn^2+^ (Figure 3C). In contrast, for WT, A99T, and D128N mutant enzymes, replacement of Mg^2+^ with Mn^2+^ led to decreased activity, similar to what was reported earlier for the WT enzyme^5^ (Figure 3C). Titration of Mn^2+^ and Mg^2+^ with P81L and WT enzymes revealed that at a 1:1 ratio (ATP/metal ion), the P81L mutant did not show any activity with Mg^2+^, but with Mn^2+^, there was a detectable activity. At a 1:3 ratio (ATP/metal ion), the P81L enzyme showed maximal activity with Mn^2+^, which was more than three times higher than that with Mg^2+^. At greater than 10 times excess of metal ions, the P81L mutant showed higher activity with Mg^2+^. For the WT enzyme, the activity was higher in the presence of Mg^2+^ at all concentrations (Figure 3D). This suggests that divalent metal ions may not only act as cofactors for enzyme catalysis but also interact with the protein and thus affect catalysis.

The influence of NaCl on the TMPK activity was also tested. Increasing NaCl concentrations had no effect on the WT TMPK activity but caused an approximately 30% reduction of the activity of the P81L and A99T mutant enzymes at the highest NaCl concentration used (Figure 3E). These results suggested that the salt-sensitive and Mn^2+^-dependent TMPK activity detected in patient-derived fibroblasts (TMPK^mut^) most likely originated from the P81L mutant enzyme.

Since both P81L and D128N are expressed in the patient-derived TMPK^mut^ cells, we assessed whether the coexistence of these mutant enzymes affects the total TMPK activity. The recombinant P81L and D128N mutant enzymes were mixed at different ratios and their combined specific activities were determined. As shown in Table 3, when present alone, P81L had 0.25% of the WT enzymatic activity and D128N had 0.08% of the WT enzyme activity. When equal amounts of P81L and D128N were mixed, the specific activity was ∼0.2% of the WT enzymatic activity, which is approximately the level of TMPK activity detected in patient-derived fibroblasts compared with the controls. This suggests that both alleles are probably expressed equally.

**Table 3:** Specific Activities of Recombinant P81L and D128N Mutants Mixed at Different Ratios^a^

ratio	P81L:D128N (%)	D128N:P81L (%)
1:0	1033 ± 198 (0.25)	333 ± 33 (0.08)
1:1	831 ± 207 (0.20)	751 ± 175 (0.18)
1:2	607 ± 145 (0.15)	841 ± 139 (0.20)
1:5	440 ± 74 (0.11)	926 ± 156 (0.22)
1:10	360 ± 37 (0.09)	937 ± 178 (0.22)

a The activity determination was performed at 37 °C using ^3^H-TMP as the substrate (1.2 μM). Each measurement was repeated five times and data are given as mean ± SD. Unit: pmol/min/mg. Numbers in parentheses are the percentage of WT TMPK.

### Subunit Interactions

**Figure 4:**
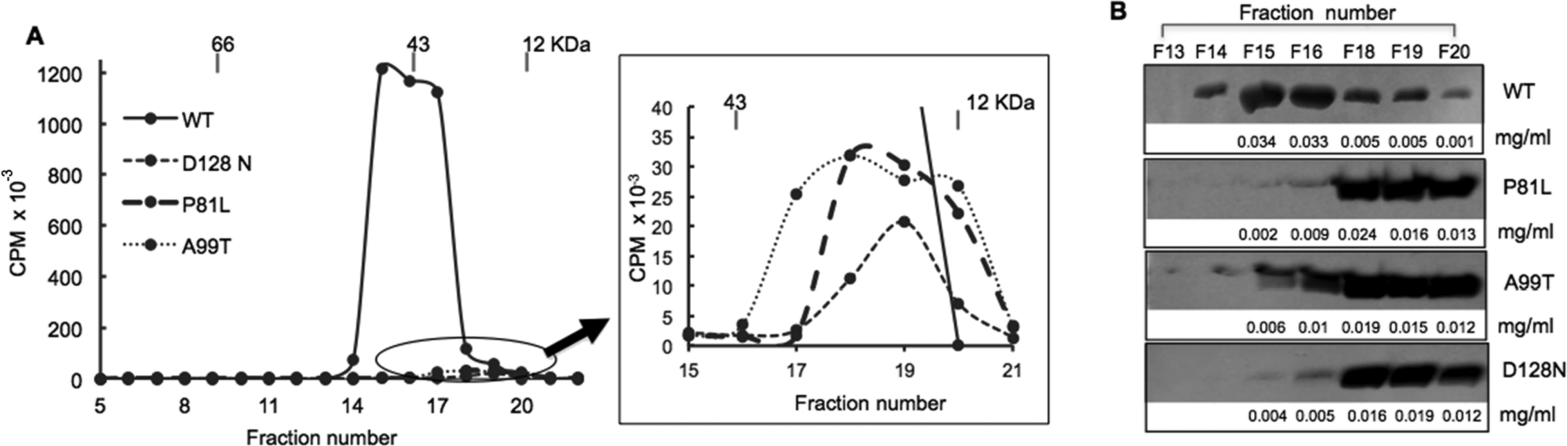
Subunit interaction of WT and mutant TMPKs. (A) Size-exclusion chromatography. Two hundred micrograms of each enzyme was analyzed. Fractions (0.4 mL) were collected and the TMPK activity in each fraction was measured (shown as CPM). Protein size markers were BSA (66 kDa), ovalbumin (43 kDa), and cytochrome C (12 kDa). (B) Western blot analysis. Ten microliters of protein from each fraction was used. The experiments were repeated at least three times for each protein; one representative image is shown. The protein concentrations in each fraction are indicated under the Western blot image.

In the crystal structure, human TMPK is in a dimer form.^6^ In order to understand the impact of mutations on TMPK catalysis, we studied the subunit interactions of these enzymes by using size-exclusion chromatography. As shown in Figure 4, WT TMPK was eluted predominantly in the dimer form (in fractions 15 and 16), as judged by activity measurements. In Western blot analysis, we could also observe a minor fraction of the WT enzyme in the monomer form (in fractions 18–20) with a very low specific activity. In contrast, all mutant TMPKs were eluted mainly as monomers (in fractions 18–20), which was also confirmed by Western blot analysis (Figure 4).

### TMPK Sequence and Structure Analysis

**Figure 5:**
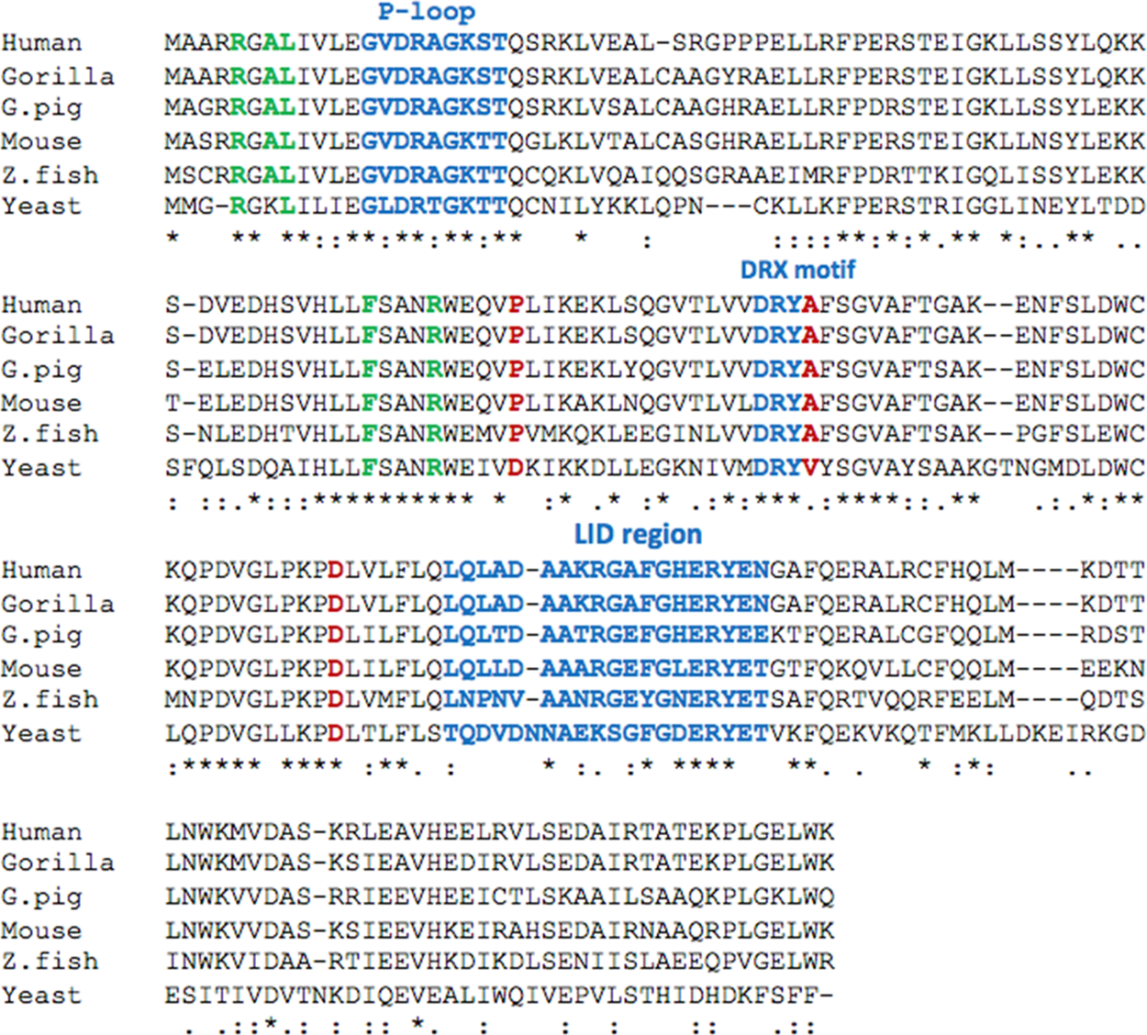
TMPK sequence analysis. Amino acid sequence alignment of thymidylate kinases from different species. Human: CAA38528.1, Gorilla: XP_004033529.1, Guinea pig (G. pig): XP_003474598.3, Mouse: NP_001099137.1, Zebrafish (Z.fish): NP_001032187.1, and Yeast: AAA35158.1. The alignment was performed at https://mafft.cbrc.jp/alignment/server/. Residues P81, A99, and D128 are marked in red. Important functional motifs, for example, P-loop, DRX motif, and LID region are marked in blue. Green-labeled residues have important functions in protein folding and ligand binding.

At the amino acid level, TMPK is highly conserved across different species, particularly for the functional domains, for example, the p-loop, the DRX motif, and the LID region (Figure 5). These point mutations identified in human patients are conserved in all vertebrates, but in yeast, the residues corresponding to human P81 and A99 were replaced by D and V, respectively (Figure 5). To elucidate the possible mechanisms of the observed drastic changes in substrate binding, catalysis, and altered subunit interactions of the TMPK mutants, we performed a structural analysis of human TMPK using PyMol software and the known structure of human TMPK in complex with dTMP, an ATP analogue, and a Mg^2+^ ion (www.rcsb.org, PDB code: 1e2f).

**Figure 6:**
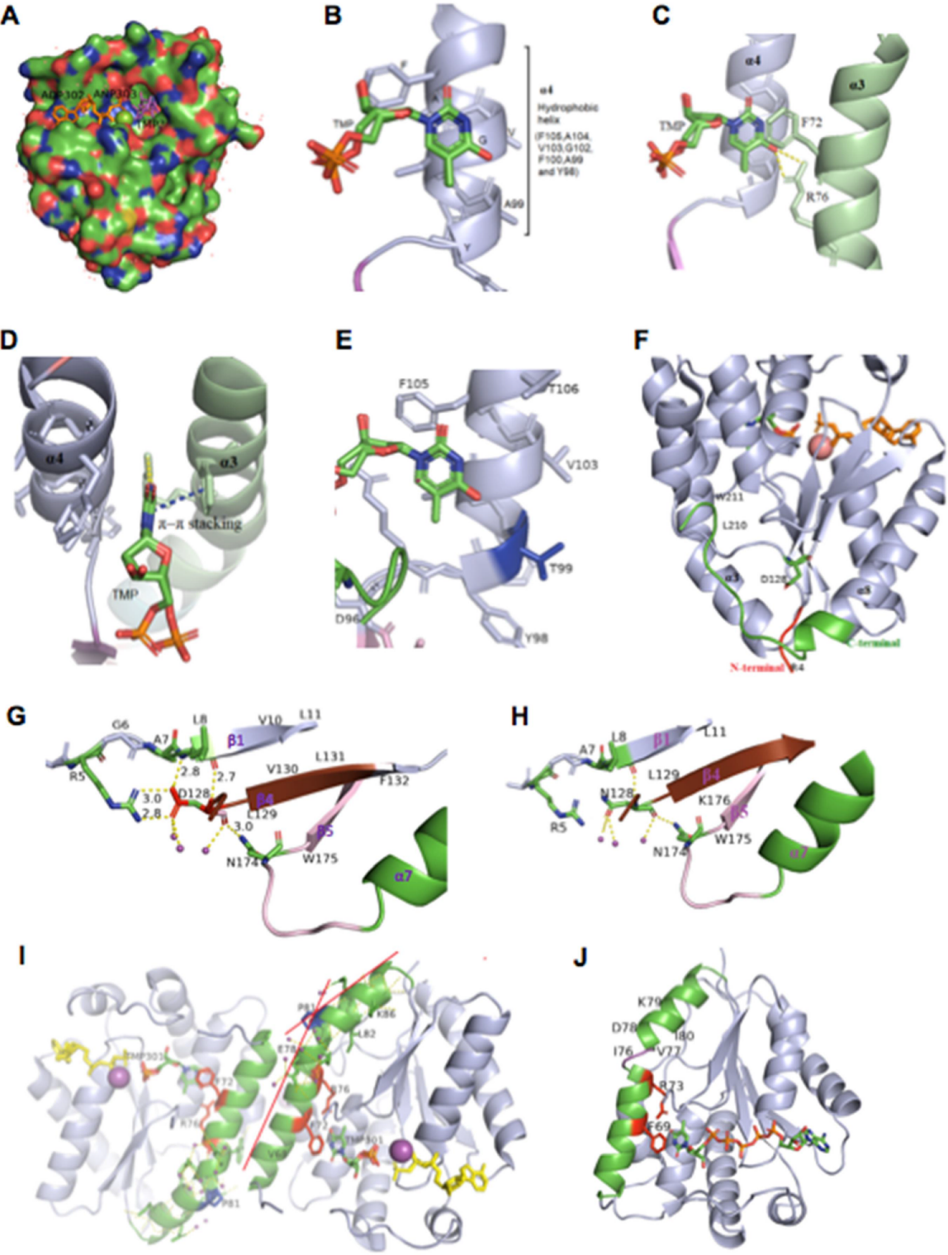
Structure analysis. The human TMPK structure (PDB code: 1e2f) was extracted from the Protein Data Bank (www.rcsb.org) and PyMol 2.3.4 was used in the analysis. (A). Overall human TMPK structure with bound substrates; dTMP (in magenta), ATP analogue ADPANP (in orange), and Mg^2+^ ion (in green); (B) α4 (in light blue) functions as a hydrophobic spine to the thymine base of dTMP (colors of the atoms are according to the element. C: green, H: gray, N: blue, O: red, and S: orange); (C) two helices position the thymine base in the middle by hydrophobic interactions. The benzyl ring of F72 on α4 (in light blue) and α3 (in lime) is in parallel with the thymine base. Two salt bridges formed between residue R72 and the thymine base further stabilize it; (D) top view of α3, dTMP, and α4. α3 and α4 are in parallel position and the thymine base is held in between; (E) A99 is replaced with T99. This changes the hydrophobic environment; (F) D128 in the 3D structure. The *N*-terminal is in red; *C*-terminal is in green; residue D128 is marked; the ATP analogue is in orange, and dTMP is colored according to the elements; (G) Interaction of D128 with neighboring residues; (H) D128 is replaced with N and changes in interaction with neighboring residues; (I). The human TMPK dimer. P81 (blue) locates at α3 (green). P81 introduces a kink resulting in a 30° bend of the helix, which directs R76 and F72 to the ligand TMP; and (J) yeast TMPK monomer structure (PDB code: 3TMK). No Pro is present in the yeast sequence. Therefore, the corresponding helix in human TMPK (e.g., helix α3 (green)) in yeast consists of two shorter helices connected by a loop structure (red color), which forms the right angle. Thus, residues R73 and F68 could interact with the thymine base in the same manner as in human TMPK.

In the WT human TMPK structure, the binding pocket for dTMP is buried while the ATP binding site is exposed, with the Mg^2+^ ion lying between them (Figure 6A). As shown in Figure 6B–D, two α helices, that is, α4 (in light blue) and α3 (in lime), are needed to position the thymine base; this is due to the hydrophobic residues Y105, A104, V103, G102, F100, A99, and Y98 on α4 forming a hydrophobic environment for the thymine base (Figure 6B). The side chain of F72 on α3 contributes with π–π stacking to the thymine base within 3.5 Å (the cutoff of π–π stacking is 4 Å) (Figure 6D). The side chain of R76 forms two polar contacts with the oxygen atom of the carbonyl group on c4 of the thymine base at ideal distances of 2.7 and 3.3 Å (Figure 6C). Thus, the residues on α3 and α4 play an important role in dTMP binding (Figure 6C,D).

A systematic study by Pace and Scholtz^7^ suggested that the propensity of amino acids to form helices is determined by their conformational entropy, that is, the energy required for proper folding. Ala has a helix propensity value of 0 kcal/mol, and the helix propensity value of Thr is 0.66 kcal/mol. Therefore, to form a helix, Thr needs higher energy than Ala, in particular, when the nearby residues have already relatively high helix propensity (F100, 0.54 kcal/mol and Y98, 0.53 kcal/mol). An A99 to T99 mutation, that is, a change from a nonpolar (Ala) to a polar (Thr) residue, may introduce new polar interactions such as H-bonds, resulting in local structural alterations that disrupt substrate binding and subunit interactions, leading to a lower binding affinity and catalytic efficiency (Figure 6B,E).

D128 is located at the bottom of the β-sheet, adjacent to the *N*-terminal (in red) and *C*-terminal (in green) (Figure 6F). A stable β-sheet is critical for the TMPK active site structure. D128 stabilizes β1 and β4 through interactions with the backbone of A7 and L8 and electrostatic interactions, that is, two pairs of salt bridges with the side chain of R5 at 2.8 and 3.0 Å distances, respectively (Figure 6G). The backbone of D128 interacts with the side chain of N174, which further stabilizes β5 and α7 since N174 locates between β5 and α7 (Figure 6G). Therefore, D128 plays a key role in stabilizing the internal dynamic structure of the enzyme (Figure 6G). D128 to N128 mutation (with an uncharged side chain) leads to the loss of electrostatic attraction to R5 and disruption of the salt bridges to A7 (Figure 6H). Thus, the D128N mutation could destabilize the β-sheet, which in turn destabilizes the active site and thus reduces the binding affinity and catalytic efficiency.

P81 is located on helix α3 that forms the monomer–monomer interface of the TMPK dimer (Figure 6I). The helix α3 contains 25 residues and is the longest helix of the protein. Proline has a rigid ring structure on its side chain, and rotation is not possible. Therefore, when proline is present in the middle of a helix, it usually destabilizes the structure element or causes a kink. The latter is relevant for P81 in human TMPK, which causes a 30° bend of the helix α3 (Figure 6I).^8^ This kink makes the interaction of F72 and R76 with dTMP possible, which contributes partly to the specificity of the enzyme. The kink also makes dimer formation possible (Figure 6I). In yeast, Pro is replaced by Asp at this position and thus the kink is not present. However, residues 75–77 form a loop structure that results in two helices instead, which provides a right angle so that R73 and F69 can interact with the thymine base in the same manner as in human TMPK (Figure 6J). Replacing P81 with L81 causes the helix to lose the kink and thereby the helix may become straight, and the residues F72 and R76 will be diverged away from the dTMP binding site and also preclude dimer formation, which results in decreased substrate binding affinity and catalytic efficiency.

## Discussion

Deoxynucleoside triphosphates including dTTP are the fundamental building blocks for DNA and are synthesized through a highly regulated process catalyzed by specific enzymes, with TMPK being a key enzyme in dTTP synthesis. Using primary fibroblasts derived from a patient with compound heterozygous mutations in TMPK (P81L and D128N), we here studied the impact of these mutations on cell proliferation and dTTP synthesis. Strikingly, we found that these mutations did not affect the proliferation rate or the morphology of the fibroblasts although the TMPK activity and protein levels were extremely low. In fact, despite the low TMPK activity, the TMPK^mut^ cells have a higher dTTP synthesis capacity compared with the controls, suggesting that there is an alternative and hitherto unknown TMPK-like compensatory enzyme present, similar to the TMPK-like activity detected recently in BJ cells.^9^

Here, we expressed and characterized three mutant TMPK enzymes (P81L, D128N, and A99T) in order to clarify the impact of the respective mutations on the enzyme function. All three mutants showed very poor substrate binding affinity and drastically reduced catalytic activity, in particular the D128N mutant, compared with the WT enzyme. Using size-exclusion chromatography, we demonstrated that all three mutants are in the monomer form. This is in contrast to the WT TMPK, which is predominantly in the dimer form with a high enzymatic activity. Notably, the monomer form of the WT enzyme had very low specific activity, suggesting that dimerization is critical for efficient catalysis. In the human TMPK structure, the P81, A99, and D128 residues have no direct interaction with the substrates. However, by structural analysis we were able to explain why the single amino acid substitutions (P81L, A99T, and D128N) result in not only drastically reduced catalytic activity and decreased substrate binding affinity but also impaired dimerization.

We also show that the residual TMPK activity in the TMPK^mut^ cells and of the recombinant P81L mutant enzyme prefers Mn^2+^ to Mg^2+^ as a cofactor for catalysis, which deviates from the WT enzyme. In a kinase-catalyzed reaction, divalent metal ions such as Mg^2+^ or Mn^2+^ coordinate with the phosphoryl groups of ATP and facilitate nucleophilic attack. These metal ions may also interact with the protein and induce conformational changes.^13^ In the human TMPK, one Mg^2+^ interacts with ATP in each subunit. However, there is also a Mg^2+^ ion present in the monomer–monomer interface (PDB code: 1e2f).^6^ Although the role of this Mg^2+^ ion has not been studied, it is likely that it may interact with the protein through electrostatic interactions and thus might play a role in dimer formation. P81 is located in the middle of helix α3, which forms the monomer–monomer interface. The change in α3 caused by the P81L mutation, as described above, may alter the interaction between the two monomers with metal ions, and Mn^2+^ (with a larger radius compared with Mg^2+^) might fit better in this position and may thus facilitate dimerization, resulting in a higher activity.

The four reported human cases with TMPK mutations showed different degrees of disease severity. The patient with compound heterozygous P81L and D128 mutations died at 18 months of age, whereas the patient with the P81L mutation died at 32 months of age.^2^ The two siblings with 34 bp deletion and an A99T mutation also showed different degrees of severity and were alive at 2 respective 7 years of age at the time of study.^3^ Notably, the A99T variant TMPK has the highest activity, whereas the D128N TMPK has the lowest activity among the three mutants, which may indicate that there is a correlation between the residual TMPK activity and the severity of the diseases.

In mitochondria, dTTP needed for mtDNA synthesis can be synthesized in situ by the salvage pathway, and thus, a mitochondrial TMPK is required.^10^ In the study describing the two siblings with the A99T mutation and 34 bp deletion, the authors suggested that the mutations likely caused loss of TMPK activity, resulting in mtDNA depletion.^3^ Indeed, the lack of mitochondrial TMPK activity observed in the TMPK^mut^ cells may indicate impaired mitochondrial dTTP synthesis. However, to prove the effect of TMPK mutations on mtDNA copy number in neuronal cells, it would be necessary to evaluate patients’ tissue material or to conduct studies in transgenic animals.

At present, the mechanism behind why a deficiency in TMPK activity causes neurodegenerative disorders is not known. Neurons, unlike other cells, have two sets of extensions outward in opposite directions, that is, dendrites and axons, which are essential for neuronal communication. Mitochondria are the most abundant organelles in neurons and are located throughout axons and dendrites. Except for energy production, mitochondria in neurons play an essential role in calcium homeostasis, which is vital for synaptic function and brain cell growth.^15,16^ The loss of TMPK activity caused by genetic alterations, that is, missense mutations and deletion, in human patients may eventually lead to mtDNA depletion and mitochondrial dysfunction, which in turn can lead to dysfunctional neurons and apoptosis of developing neurons.^17,18^

Defects in enzymes involved in the pyrimidine nucleotide metabolism have profound impacts on human neuropathology.^19,20^ Except for TMPK deficiency causing neurodevelopmental disorders as described by us and others,^2,3^ deficiency in dihydropyrimidine dehydrogenase causes seizure, intellectual disability, and microcephaly;^21^ deficiency in dihydroorotate dehydrogenase leads to Miller syndrome, possibly through dysfunctional mitochondria;^22^ thymidine phosphorylase deficiency causes mitochondrial neurogastrointestinal encephalopathy;^23^ and thymidine kinase 2 (TK2) deficiency causes devastating mitochondrial DNA depletion and/or deletion diseases with neuromuscular involvement.^24,25^ However, the mechanism behind the tissue specificity of these diseases is still not well understood although tissue-specific expression of these enzymes may play an important role.^25^ Therefore, future investigations regarding the expression and distribution of these enzymes during different developmental stages and their effects on mitochondrial function are critical to answer the question why neurons are excessively vulnerable to impaired pyrimidine nucleotide metabolism.

## Conclusions

The TMPK mutations identified in human patients represent loss of function mutations but, surprisingly, the proliferation rate of the patient-derived fibroblasts carrying such mutations was normal, suggesting the existence of an alternative and hitherto unknown compensatory TMPK-like enzyme for dTTP synthesis. The present study may contribute to the understanding of basic biochemical pathways in dTTP synthesis as well as to understanding the role of TMPK in neurodegenerative diseases. Furthermore, the present study may aid in future attempts to design therapeutic interventions for diseases caused by defects in enzymes involved in the pyrimidine nucleotide metabolism.

## Materials and Methods

### Materials

Dulbecco’s modified Eagle’s medium (DMEM) and sodium pyruvate were obtained from Sigma-Aldrich; heat-inactivated fetal bovine serum (FBS) and penicillin–streptomycin were obtained from Thermo Fisher; trypsin–EDTA was obtained from the Swedish National Veterinary Institute; Bradford protein determination solution was obtained from AppliChem; DEAE filter paper (DEAE filtermat) and liquid scintillation fluid (Optiphase HiSafe 3) were purchased from PerkinElmer; and a PEI cellulose F plate was obtained from Merck group. The polyclonal antibody against human TMPK was produced by using the C-terminal peptide sequence as antigen (GenScript Inc).^9^ The antibody against beta-actin was from Santa Cruz Biotechnology. Secondary fluorescence antibodies were from LI-COR Biosciences.

### Cell Culture

The TMPK^mut^ fibroblasts were derived from the patient with compound heterozygous mutations (P81L and D128N) and Cont were derived from the patient’s mother as described earlier.^2^ BJ cells immortalized with hTERT^26^ was kindly provided by Prof. Staffan Johansson (Uppsala University, Sweden). Cells were cultured at 37 °C in a humidified incubator with 5% CO_2_ in complete DMEM media containing 10% heat-inactivated FBS, 1 mM sodium pyruvate, and 1% penicillin–streptomycin. The medium was changed when the cells reached 80–90% confluence.

### Subcellular Fractionation

Freshly harvested cells were washed twice with ice-cold phosphate-buffered saline (PBS) and then used to isolate subcellular fractions by differential centrifugation, essentially as described.^27^ The resulting subcellular fractions were stored in aliquots at −80 °C until further analysis. The protein concentrations were determined using the Bradford method and bovine serum albumin (BSA) as the standard.

### Expression and Purification of the WT and Mutant TMPKs

All mutant (P81L, A99T, and D128N) TMPKs were cloned into the pET-14b vector with an *N*-terminal fusion 6xHis tag and expressed in *E. coli* strain BL21 (DE3) pLysS. The recombinant enzymes were expressed and purified as previously described.^28^ WT TMPK was run in parallel for comparison. The purity of the proteins was >95% as judged by the SDS-PAGE analysis.

### Western Blot Analysis

Appropriate amounts of cell extracts or recombinant TMPKs were resolved on 12% SDS-PAGE gels. After gel electrophoresis, the protein bands were transferred to a PVDF membrane. After blocking, the membranes were probed with a primary antibody against human TMPK, and the TMPK protein bands were visualized using a fluorescence secondary antibody and the Odyssey system (LI-COR Biosciences).

### Radiochemical Enzyme Assays

TK and TMPK activities were determined as described previously.^28,29^ Briefly, the TK reaction mixture contained 10 mM Tris/HCl pH 7.6, 5 mM MgCl_2_, 0.5 mg/mL BSA, 5 mM dithiothreitol (DTT), 1 mM ATP, 2 μM ^3^H-dT, 28 μM dT, and an appropriate amount of proteins. For TMPK activity determination, ^3^H-dTMP was used as the substrate.^29^ The reaction mixtures were incubated at 37 °C for a total of 30 min. At different time points, aliquots of the reaction mixture were spotted onto the DEAE filter paper. After drying, the unreacted substrate was washed away with ammonium formate (1 mM for the TK assay and 50 mM for the TMPK assay). The products were eluted with 0.5 mL of HCl (0.1 M) and KCl (0.2 M). After addition of a scintillation fluid, the radioactivity was counted (Tri-Carb, PerkinElmer).

### Coupled Spectrophotometric Assay

Steady-state kinetic studies of the WT and TMPK mutants were carried out using a coupled spectrophotometric method essentially as previously described.^28,30^ The reaction mixture contained 10 mM Tris/HCl pH 7.6, 5 mM MgCl_2_, 5 mM DTT, 0.5 mM phosphoenolpyruvate, 0.1 mM NADH, 4 units/mL pyruvate kinase, 4 units/mL lactate dehydrogenase, ATP (1 mM or at variable concentrations), and dTMP (100 μM or at variable concentrations). The reactions were started by the addition of the TMPK enzymes, and the rate of NADH oxidation was monitored at 340 nm using a spectrophotometer for 2 min at room temperature (21 °C). All assays were repeated at least three times, and the results are given as mean ± SD. The kinetic parameters were calculated by fitting the initial velocity data to the Michaelis–Menten equation, *V*_0_ = *V*_max_ [*S*]/(*K*_M_ + [*S*]).

### ^3^H-dT Uptake and Metabolism

A total of 300,000 cells were seeded in T25 cell culture flasks for 24 h, and then 0.5 μM ^3^H-dT was added to the cell culture. The cells were then incubated for 10 h. The cells were harvested and the media was collected. The cells were washed three times with ice-cold PBS, and the nucleotides were extracted with 10% PCA on ice. One microliter of the soluble nucleotide extracts or the cell culture media was spotted on a thin-layer chromatography (TLC) plate and then developed in 0.2 M sodium dihydrogen phosphate buffer.^31^ After developing, the TLC plate was dried and cut into 1 cm in length, transferred into vials, and eluted with 0.5 mL elution buffer (0.2 M KCl and 0.1 M HCl) for 25 min on a shaker at room temperature. The radioactivity was counted after the addition of the scintillation fluid. ^3^H-dT, ^3^H-dTMP, ^3^H-dTDP, and ^3^H-dTTP were used as the standards.

### DNA Extraction from Fibroblasts

Freshly harvested cells were washed three times with ice-cold PBS, and then the cell pellet was resuspended in 500 μL buffer (100 mM Tris/HCl, pH 7.4, 5 mM EDTA, 0.25 mg/mL proteinase K, 0.1% SDS, and 200 mM NaCl) and incubated at 50 °C overnight. The mixture was then centrifuged at 16,000 ×*g* for 20 min at 4 °C and the supernatants were transferred into new tubes. Next, 300 μL of ice-cold isopropanol was added and incubated for 1 h on ice and then centrifuged for 20 min at 16,000 ×*g* at 4 °C. The pellet was washed with ice-cold 70% ethanol and then air-dried. Water (100 μL) was added to the pellet to dissolve the DNA. Finally, 10 μL of DNA was mixed with the scintillation fluid and the radioactivity was counted.

### Size-Exclusion Chromatography

A Superdex G200 column (GE Healthcare) was pre-equilibrated with a buffer containing 10 mM Tris/HCl, pH 7.6, 100 mM NaCl, 5 mM MgCl_2_, and 5 mM DTT. BSA (66 kDa), ovalbumin (43 kDa), and cytochrome C (12 kDa) were used as protein size markers. WT and mutant TMPK proteins (200 μg) were injected into the column and eluted with the same buffer. The flow rate was 0.4 mL/min. Fractions of 0.4 mL were collected. Each fraction was subjected to Western blot and enzyme activity analyses. The protein concentrations were determined using the Bradford method with BSA as the standard.

### Structure Analysis

The human TMPK structure with bound ligands TMP, ATP analogue (ADPANP), and Mg^2+^ ion was extracted from the Protein Data Bank (www.rcsb.org, PDB code: 1e2f). The structure was analyzed using PyMol 2.3.4 software. The rotamer of an amino acid with fewest steric clashes was chosen in each mutagenesis. Due to the limitation of PyMol, energy minimization of the protein structure is not taken into consideration in the mutagenesis.

### Statistical Analysis

All data were analyzed using Microsoft Office Excel 2010 and GraphPad Prism software. Statistical comparisons were performed using a two-tailed *t*-test. All data were derived from at least three independent experiments and are presented as the mean ± S.D.

## Supporting Information Available

Thymidine kinase activity and kinetic study of the D128N mutant (PDF)

## Accession Codes

Accession ID for human TMPK: UniProt: P23919 (KTHY_HUMAN). NCBI: CAA38528.1.
