# Supplementary material for: Biochemical Characterizations of Human TMPK Mutations Identified in Patients with Severe Microcephaly: Single Amino Acid Substitutions Impair Dimerization and Abolish Their Catalytic Activity
Source: ACS Omega. 2021 Dec 6;6(49):33943–52. doi: 10.1021/acsomega.1c05288 (PMC8679000; doi:10.1021/acsomega.1c05288)
Supplement: Supplementary file 1 — ao1c05288_si_001.pdf [file ao1c05288_si_001.pdf]

SUPPORTING INFORMATION

Biochemical characterizations of human TMPK mutations identified in patients with severe microcephaly: Single amino acid substitutions impair dimerization and abolish its catalytic activity

Junmei Hu Frisk[a], Jo M. Vanoevelen[b], Jörgen Bierau[b], Gunnar Pejler[a,c], Staffan Eriksson[a], and Liya Wang[a*]

[a]Department of Anatomy, Physiology and Biochemistry, Swedish University of Agricultural Sciences, Uppsala, Sweden

[b]Department of Clinical Genetics, Maastricht University Medical Centre+ and GROW school for oncology and developmental biology, Maastricht, The Netherlands.

[c]Department of Medical Biochemistry and Microbiology, Uppsala University, Uppsala, Sweden

*Corresponding author: liya.wang@slu.se

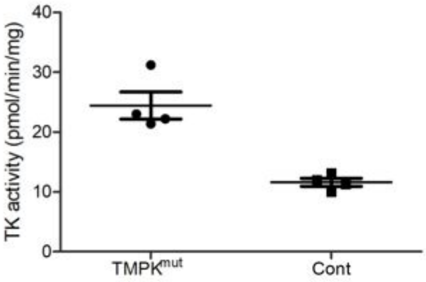

Figure S1. Thymidine kinase activity. Cell lysates from TMPK^{mut} and Cont were used for TK activity measurement using ^{3}H-dT as substrate.

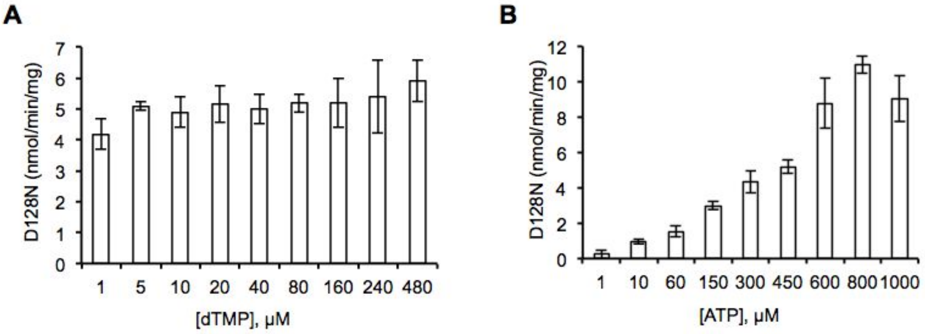

Figure S2. Kinetic study of D128N mutant. The D128N mutant activity determined with (A) variable dTMP concentration (1-480 μM) and fixed ATP (1 mM) concentration, and (B) variable ATP concentration (1-1000 μM) and fixed dTMP concentration (100 μM).
